# Advancing interdisciplinary research in head and neck cancer through a multicenter longitudinal prospective cohort study: the NETherlands QUality of life and BIomedical Cohort (NET-QUBIC) data warehouse and biobank

**DOI:** 10.1186/s12885-019-5866-z

**Published:** 2019-08-05

**Authors:** I.M. Verdonck-de Leeuw, F. Jansen, R. H. Brakenhoff, J. A. Langendijk, R. Takes, C. H. J. Terhaard, R. J. Baatenburg de Jong, J. H. Smit, C. R. Leemans

**Affiliations:** 1 Department of Otolaryngology-Head and Neck Surgery, Cancer Center Amsterdam, Amsterdam UMC, Vrije Universiteit Amsterdam, PO BOX 7057, 1007 MB Amsterdam, The Netherlands; 2 Department of Clinical, Neuro and Development Psychology, Vrije Universiteit Amsterdam, Amsterdam Public Health Research Institute, Amsterdam, The Netherlands; 3 Department of Radiation Oncology, University Medical Center Groningen, University of Groningen, Groningen, The Netherlands; 4 Department of Otolaryngology-Head and Neck Surgery, Radboud University Medical Center, Nijmegen, The Netherlands; 5 Department of Radiation Oncology, University Medical Center, Utrecht, The Netherlands; 6 Department of Otolaryngology and Head and Neck Surgery, Erasmus Cancer Institute, ErasmusMC, Rotterdam, the Netherlands; 7 Department of Psychiatry, Neuroscience Campus Amsterdam and Amsterdam Public Health Research Institute, Amsterdam UMC, location VU University Medical Center, Amsterdam, The Netherlands

**Keywords:** Head and neck cancer, Survival, Health related quality of life, Symptoms, Toxicity, Data warehouse, Biobank, Cohort study, Caregivers

## Abstract

**Background:**

Worldwide, over 500,000 people are diagnosed with head and neck cancer each year, a disease with major impact on life expectancy and quality of life. The purpose of the Netherlands Quality of life and Biomedical Cohort study (NET-QUBIC) is to advance interdisciplinary research that aims to optimize diagnosis, treatment, and supportive care for head and neck cancer patients and their informal caregivers.

**Methods:**

Using an extensive assessment protocol (electronic clinical record form, patient reported outcome measures and fieldwork (interviews and physical tests)), clinical data and data on quality of life, demographic and personal factors, psychosocial (depression, anxiety, fatigue, pain, sleep, mental adjustment to cancer, posttraumatic stress), physical (speech, swallowing, oral function, malnutrition, physical fitness, neurocognitive function, sexual function), lifestyle (physical activity, nutrition, smoking, alcohol, drugs), and social factors (social function, social support, work, health care use, and costs) are collected and stored in the data warehouse. A longitudinal biobank is built with tumor tissue, blood and blood components, saliva samples, and oral rinses. An infrastructure for fieldwork and laboratory protocols is established at all participating centers. All patients fill out patient reported outcome measures before treatment and at 3, 6, 12, 24, 36, 48, and 60 months follow-up. The interviews, physical tests and biological sample collection are at baseline and 6, 12, and 24 months follow-up. The protocol for caregivers includes blood sampling and oral rinses at baseline and a tailored list of questionnaires, administered at the same time points as the patients. In total, 739 HNC patients and 262 informal caregivers have been included in 5 out of the 8 HNC centers in the Netherlands.

**Discussion:**

By granting access to researchers to the NET-QUBIC data warehouse and biobank, we enable new research lines in clinical (e.g. treatment optimization in elderly patients), biological (e.g. liquid biopsy analysis for relapse detection), health related quality of life (e.g. the impact of toxicity on quality of life), and interrelated research (e.g. health related quality of life in relation to biomarkers and survival).

## Background

Worldwide, more than half a million people per year are diagnosed with head and neck cancer (HNC) [1], a disease with major impact on the patient but also on their partner, and family. In the Netherlands, almost all HNC patients are treated in specialized HNC centers. HNC survival rates in the Netherlands are more favorable compared those in other European countries [2], which can in part be explained by this centralization of treatment and care. However, there is still room for improvement, not only with respect to survival but also regarding symptom management and health related quality of life (HRQOL) [3–5].

Previous research over the past decades provided convincing evidence that cancer patients in general have to deal with various physical, psychological, and social side effects of cancer and cancer treatment, negatively affecting HRQOL. In HNC patients, specific stressors as oral dysfunction (e.g. xerostomia) and related swallowing and speech impairment and malnutrition often lead to emotional distress as depression and anxiety. This previous research also showed considerable variation between patients: some patients are at risk for poor HRQOL, while others are protected [6–18]. Cancer does not only have a major impact on HRQOL of HNC patients, but also on HRQOL of their informal caregivers [19–28]. Limited data exists on the supportive care needs of HNC patients and their caregivers, and these needs may depend on the type of HNC and the time point of the cancer illness trajectory [29–32]. In addition to the impact on patients and caregivers, cancer may also put burden on society. HNC patients have higher medical care consumption and are more likely to be unemployed than other cancer patients [33–38].

In HNC patients, associations between HRQOL and survival have been found. Factors influencing survival (e.g. age at time of diagnosis, tumor stage, metastasis, and comorbidity) have impact on HRQOL. Additionally, HRQOL has prognostic value for survival in HNC cancer patients, independently from known predictors as sociodemographic and clinical parameters [39–52]. However, the association between HRQOL and survival is complex. Empirical evidence suggests that tumor- and patient-related biomarkers of endocrine, immune, and autonomic (dys)function are associated with both HRQOL and survival [53–58]. Biomarkers of neuroendocrinological and neuroimmunological function may play a role in the association between HRQOL and survival. Neuroendocrinological markers include the activation of the HPA-axis resulting in increased secretion and flattened circadian rhythm of cortisol, and specific hormone levels may impact resistance mechanisms during treatment. Neuroimmunological explanations include increased immune responses and increased levels of pro-inflammatory cytokines (interleukin (IL)-1, IL-6 and TNF-a). Comprehensive insight in all these factors assessed in a standardized manner in large study populations is necessary to unravel these complex associations.

Despite the existing body of evidence, there is also an unmet need for better understanding of all aspects of HRQOL in the context of increasing long-term survival and growing attention for cancer survivorship [32, 59–61]. This paper describes a project that aims to collect longitudinal data and answer some of these questions. In addition, data is stored in a national data warehouse that can be disseminated to other researchers. The longitudinal design of the study enabled the longitudinal collection of biological samples and storage in a biobank of this large prospective cohort of HNC patients and their informal caregivers. The study is indicated as the NETherlands Quality of life and Biomedical Cohort study in head and neck cancer (NET-QUBIC) (www.kubusproject.nl). In this protocol paper, sociodemographic and clinical characteristics of the study sample of 739 patients and 262 informal caregivers are provided. Also, the key components of the NET-QUBIC project are described: the study population (including sample size calculation), the comprehensive electronic case report form (eCRF), the extensive outcome assessment protocol, biobanking protocols and quality controls, data management (collection and storage), and data and sample dissemination procedures (including legal issues). This paper thus also facilitates other consortia planning to set up a large prospective cohort study such as NET-QUBIC.

## Methods/design

### Aim, design, and setting

The purpose of the Netherlands Quality of life and Biomedical Cohort study in HNC (NET-QUBIC) is to advance interdisciplinary research that aims to optimize diagnosis, treatment, and supportive care targeting HNC patients and their informal caregivers. NET-QUBIC is designed as a longitudinal observational cohort study. In parallel to the data collection, is the establishment of a biobank.

### Study population

Inclusion criteria in NET-QUBIC are: newly diagnosed squamous cell carcinomas in the head and neck (oral cavity, oropharynx, hypopharynx, larynx, unknown primary; all stages); age > 18 years; treatment with curative intent; all treatment modalities (surgery, radiotherapy, chemotherapy and combinations); able to write, read, and speak Dutch. Exclusion criteria are: other tumors in the head and neck (e.g. lymphoma, skin malignancies, thyroid cancer); patients unable to understand the questions or test instructions; and severe psychiatric co-morbidities (i.e. schizophrenia, Korsakoff’s syndrome, severe dementia), or unable to understand informed consent. Eligible patients are treated according to the current standard in the participating centers and that have been defined in national guidelines on diagnosis, treatment, and follow-up care. Via the patient, the spouse or another family member or lay caregiver (informal caregiver) is asked to participate in this project.

### Sample size calculation

The sample size calculation is based on the primary research question, i.e. to describe the course of HRQOL over time. We aim to detect a difference over 60 months of 4 points change on the global HRQOL scale of the European Organization for Research and Treatment of Cancer (EORTC) quality of life questionnaire QLQ-C30 between categories of relevant variables (for example between patients with and without depression), using a residual standard deviation of 10 points within categories, and using an α of 0.05 and a power (β) of 0.80. For the dependency of the 5 repeated measures in the study, we assume an intraclass correlation coefficient of 0.50. This results in a total sample size of 462. Taking into account study attrition of 60% (35% dropout due to mortality and 25% due to other causes), we aim to include 739 HNC patients. In addition, informal caregivers when available during intake will be included. Based on our previous research indicating that 70% of HNC patients have a caregiver and 50% willing to participate, we expect to include 258 informal caregivers.

### Case report form

An electronic Case Report Form has been built (OpenClinica) and information is retrieved from medical records. Consensus was reached on the data items to be collected, and changes during the study such as TNM8 impacted the data structure. General clinical information as well as tumor characteristics, detailed treatment information, and pathology data are collected by trained researchers. General information includes incidence date, age, sex, physical performance, performed diagnostics, ACE-27 comorbidity score and weight loss prior to treatment. Tumor characteristics include tumor subsite, lateralization, stage, and lymph node metastasis, according to the TNM7 and TNM8 classification, and in oropharyngeal tumors HPV status and type of HPV test. Information on treatment encompasses a question on type of treatment, followed by more detailed questions per treatment option.

For surgery, information on type of resection, reconstruction, type of neck dissection (including details about removed levels and nonlymphatic structures), tumor-free margins, extended morphology of the tumor, grade of differentiation, number of nodes removed, number of tumor-positive nodes, extranodal spread.

For radiotherapy, information on type of radiotherapy (primary, postoperative or other), technology use, boost methods, and start and end date of radiotherapy is collected. In addition, for patients treated with primary definitive radiotherapy, information on total dose, fraction dose and fractions per week delivered to primary tumor and N+ neck is collected, as well as total dose, fraction dose, number of fractions per week, and radiotherapy duration delivered to elective lymph nodes. For patients treated with postoperative radiotherapy, information on total dose, fraction dose, number of fractions per week and the overall treatment of radiation to the high and intermediate risk and elective target volumes is collected.

For chemotherapy, information on type of chemotherapy (induction, concomitant chemoradiation, concomitant bioradiation, postoperative chemotherapy, or other), used cytostatic agents, chemotherapy scheme, completion of chemotherapy or reason for non-completion, start date, final dose (mg/m2), and information on adverse events is collected.

### Outcome assessment protocol

Data are collected according to an assessment protocol (patient reported outcome measures (PROMs) and fieldwork) and subsequently stored in the data warehouse. PROMs target demographic (age, gender, socio-economic status, marital status, literacy), personal factors (personality, coping style), HRQOL, psychological (depression, anxiety, fatigue, pain, sleep, mental adjustment to cancer, posttraumatic stress), physical (speech, swallowing, physical fitness, neurocognitive function, sexual function), social (social function, social support, work, health care use, and costs), and lifestyle (physical activity, smoking, alcohol, drugs) factors. An overview of the PROMs is provided in Table 1.

**Table 1:**
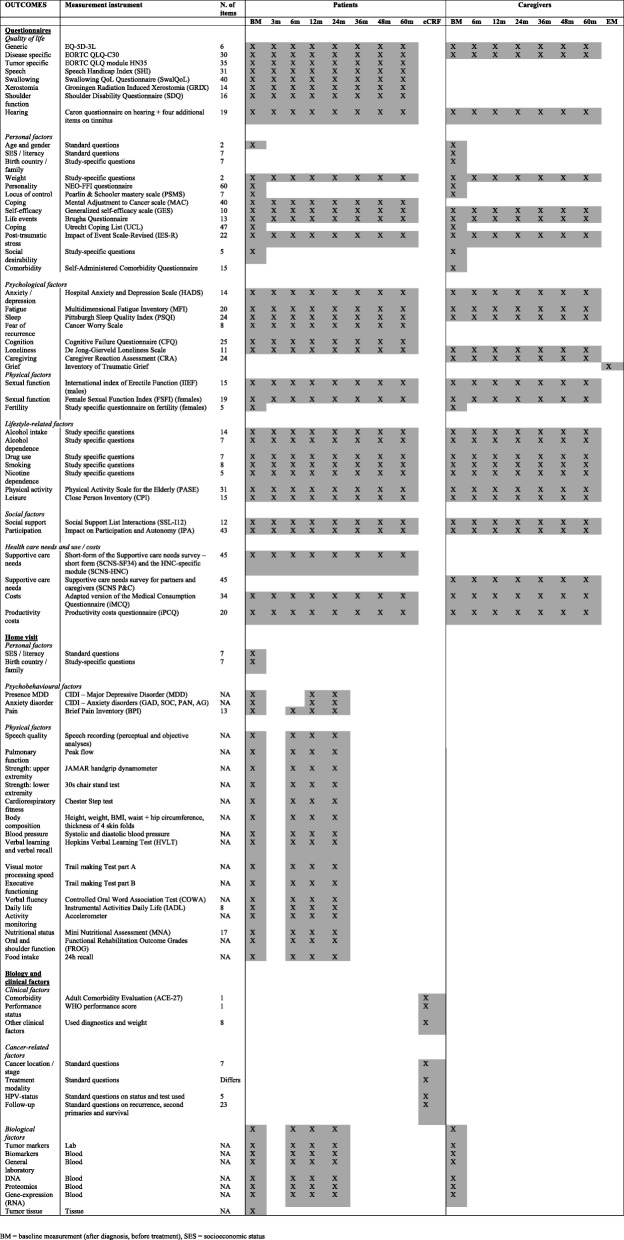
Overview of all NET-QUBIC outcome measures and assessment times

The interviews target the presence of depression and anxiety (World Health Organisation (WHO)-Composite International Diagnostic Interview version (CIDI), Nutritional status (Mini Nutritional Assessment (MNA) and Dietary intake (24-h recall). Physical tests include assessment of body composition (height, weight, weight loss, circumferences (waist, hip, and upper arm), and skinfolds), systolic and diastolic blood pressure, cardiorespiratory fitness (step test), upper (hand grip test) and lower (chair test) extremity muscle strength, and neurocognitive function (memory: Hopkins Verbal Learning Test; verbal fluency: Controlled Oral Word Association; visual-motor scanning speed: Trail Making Test Part A; executive functioning: Trail Making Test Part B). Objective levels of physical activity are assessed using an accelerometer (ActiTrainer). Oral function is assessed via the Functional Rehabilitation Outcome Scale (FROG), and assessment of trismus and dentures. Digital speech recordings are made via a standardized procedure enabling speech quality analyses. An overview is provided in Table 1.

### Biobanking

In NET-QUBIC frozen tissue, blood components and oral rinses are collected at baseline, 6, 12, and 24 months. Formalin-fixed paraffin-embedded biopsies are routinely available from the pathology archives, but biobanking of frozen samples demands collection of extra biopsies. When patients are surgically treated, the resected specimen is sampled, but in all other cases additional biopsies are collected. Generally, 1 to 3 biopsies are taken, depending on the size of the tumor. These multiple biopsies increase available tissue amounts and allow studying intratumor heterogeneity. All oropharyngeal tumors are tested for HPV virus infection as routine using p16 immunostaining followed by an HPV DNA PCR for the p16-immunopositive cases. The tissue biopsies are snap frozen and stored in liquid nitrogen.

Oral rinse sampling is performed and saliva sampling includes four saliva samples per assessment: at the time of awakening, 30 min post-awakening, 60 min post-awakening, and at 22:00 h. Afterwards, the subjects are asked to return the samples by postal mail to the coordinating research center. After receipt, salivettes are centrifuged at 2000 g for 10 minutes, aliquoted and stored at − 20 °C.

Blood samples are obtained to assess parameters of cardiovascular, main organ function, immune function, and metabolic syndrome using routine assays. Additional blood samples are processed in components and stored at − 80 °C. An EDTA sample is sent to the coordinating center and DNA isolated and stored. A PaxGene tube of blood for RNA profiling is collected and stored at − 20. A serum sample is collected, centrifuged, the supernatant aliquoted and stored at − 20 °C. Four additional EDTA samples are collected and used for isolation and storage of plasma, blood platelets, cryopreserved Peripheral Blood Mononuclear Cells and pelleted Polymorphonuclear leukocytes. A Standard Operating Procedure (SOP) forms the basis to collect, isolate and store these components. The consented SOP was introduced to the participating centers and for quality control, samples were isolated at every center from healthy volunteers, and the sample performance tested at a central laboratory (Amsterdam UMC) in functional (Antibody-dependent cellular cytoxity of PBMCs) and molecular assays (all other samples). Quality control was sparsely budgeted and thus limited, but revealed that all samples from all centers could be assayed well. Most variable was the quality and functional activity of cryopreserved PBMCs as expected, but with proper experimental controls also these samples are evaluable.

### Data management: planning, collection and storage

#### Planning

**Fig. 1:**
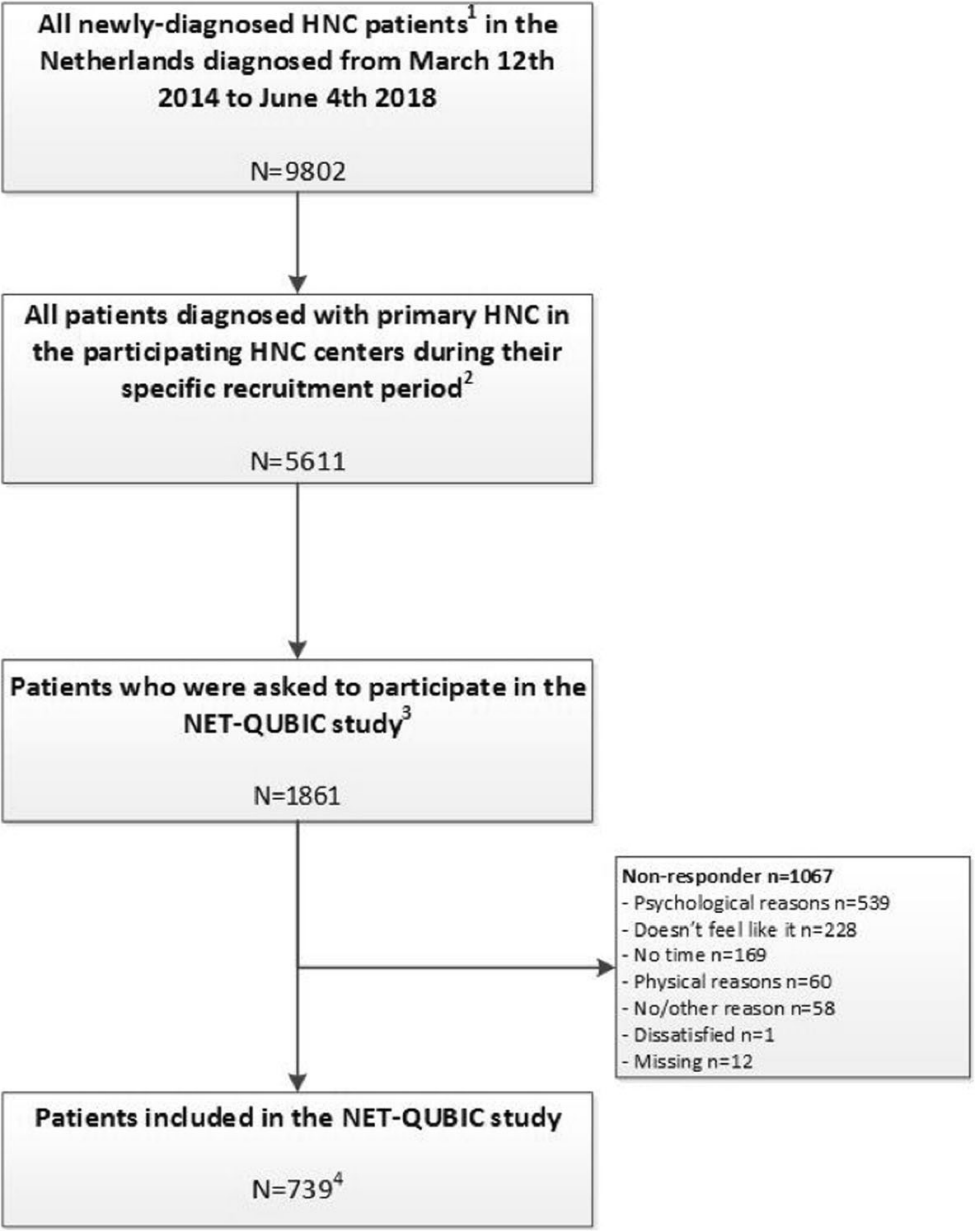
Flowchart of all eligible HNC patients and reasons for non-participation

All patients fill out PROMs before treatment and at 3, 6, 12, 24, 36, 48, and 60 months follow-up. Interviews, physical tests and biological sample collection are assessed at baseline and 6, 12, and 24 months follow-up only (because of budget restraints) (Fig. 1). The protocol for caregivers includes blood samples and oral rinse at baseline and a tailored list of questionnaires, administered at the same time points as the patients.

In total, 739 HNC patients and 262 informal caregivers have been enrolled. Data and sample collection is still ongoing and will continue to 2023 (60 months follow-up). Participants are informed about the study progress by newsletters and the website (kubusproject.nl). There is prompt and adequate response to any question or letter from the participants. The patient and the general practitioner are informed on the home test results regarding e.g. body mass index, blood pressure or blood results, and in case of deviant test scores also the attending head and neck surgeon, radiation oncologist and/or medical oncologist (for care-givers, only their general practitioner).

#### Data collection

Data and sample collection take place at home visits or at the hospital, and consists of PROMs, an interview and functional tests, and collection of biological samples. PROMs are sent to the patients by postal mail. During home visits, neurocognitive tests, physical tests and a face to face interview take place, and blood samples are drawn. Tubes to collect saliva, and the accelerometer are given to the patients after the home visit. After completion of the measurements, patients send the tubes and accelerometer to the researcher by postal mail with the envelope provided. Patients are allowed not to complete all three components (PROMs, interview/tests, biobanking) if this is too much burden, or for logistic reasons (e.g. time too short between diagnosis and start of treatment).

Research assistants conduct the field work, and manage the data collection. All data are coded directly after being collected and are entered into the NET-QUBIC data warehouse to ensure accuracy and completeness of the data. The field workers are extensively trained and supervised by a coordinating research nurse. Data management is coordinated at the Department of Otolaryngology / Head & Neck Surgery of Amsterdam UMC location VUmc, and GGZInGeest in Amsterdam. A special team of data managers takes care of data quality, data archiving and the creation of variables and scales. The NET-QUBIC Data Warehouse makes use of Open Clinica (collection of multisite case record forms) and Blaise (face to face data collection). This Data Warehouse is used to collect and store cancer and treatment data, outcome, PROMs, results of medical examination and function tests, and biobank data. Also, the logistics for project management are structured by means of this system.

#### Data storage

Data and samples are stored via FAIR principles: Findable, Accessible, Interoperable, Re-usable (www.force11.org). Findable data: Data are described on the NET-QUBIC project website (www.kubusproject.nl). Accessible data: The collection and integration of large amounts of personal, biological, genetic and diagnostic information precludes open access to the NET-QUBIC research data. In the section Data and sample dissemination (below) is described how the data are made available for the research community. Interoperable data: All data is encoded by a single participant number that includes an institute code. This allows exchange of all collected data, but adding new clinical data requires that the codes at the participating institutes are broken. Re-usable data: Validated longitudinal data from the 739 patients and 262 caregivers, as fixed datasets will be available for re-use. The storage time is 15 years as the legal minimum for clinical data.

#### Data and sample dissemination

Data and sample releases will be organized 1 to 2 times a year and will be announced on the project website (www.kubusproject.nl). Usage of NET-QUBIC data and/or biomaterials is regulated in the NET-QUBIC Data and Biomaterial Access Policy, and in the NET-QUBIC Publication policy (study procedures available via the website). In brief, access is provided as follows: A single-entry portal to all activities is provided on the NET-QUBIC website. The proposals can be submitted via an on-line application form. The NET-QUBIC team oversees the projects from evaluation, to experiment, to report after completion. Questionnaires are sent to users (i.e. 1 year following access provision) for feedback on the access service and to summarize the results (including patents and publications), enabling an evaluation of the outcome of the projects. An applicant will first choose the type of support and sample/data that they need for their proposed projects. The forms will be filled out by the applicant, and submitted to the NET-QUBIC Access Board for review. Transparency, fairness and impartiality are key objectives for the evaluation of the project ideas and project applications. Applications are judged on (i) scientific merit / excellence, and (ii) expected scientific, societal and economic impact, both carrying equal weight. While judging research protocols, there will also be emphasis on overlap between protocols (e.g. PhD-trajectories) and in case of overlap, researchers are encouraged to collaborate. Evaluation reports will be provided for all projects, including those that fail. These reports will provide constructive feedback from the evaluators that will help researchers to improve their projects. When a research protocol is granted, the data are made available by a fully automatic procedure by means of a protected website tool for researchers to download the requested data. For use of biobank samples, the research methods and requested amounts are critically reviewed and projects combined whenever possible. Before biobank samples are released, the research protocols are reviewed by the Institutional Review Board on Biobanking at the coordinating center. The participating centers agreed to this approach. Data and samples are accessible for external parties by collaboration with one of the Access Board members of the participating institutes.

A Data Transfer Agreement (DTA) or a Material and data Transfer Agreement (MTA), all according to national and European Law and Regulations as the European General Data Protection Regulation (GDPR) will be signed by the legal representative of the institute of the researcher and the PI. It also contains an Intellectual Property Strategy. The Technology Transfer Office in Amsterdam (IXA) and the legal representatives of the Board of Directors of all participating institutes have been actively involved in developing the Research Agreement, the data and biomaterial access and publication policies, and DTA/MTA. Research data obtained from new studies (e.g. new biomarkers) will be brought into the data warehouse for use in future studies.

### Study cohort

The flow diagram is shown in Fig. 1. Recruitment took place from March 2014 to June 2018. During this study period, in all 8 HNC centers in the Netherlands 9802 patients were treated for oral cavity, oropharynx, hypopharynx, or larynx cancer, or unknown primary tumors (the included HNC subsites in NET-QUBIC), of which 5611 in the NET-QUBIC centers (5 out of the 8 HNC centers). In the 5 NET-QUBIC centers, 2777 patients were screened for eligibility of which 1861 patients were eligible and approached for participation (see also Fig. 1). Reasons for not asking patients to participate were not systematically documented, but frequently mentioned reasons were that a) time between diagnosis and start of treatment was so short that obtaining informed consent before treatment was not possible, b) patients were considered too fragile to participate, or c) recruiters overlooked patients for participation.

Of the 1861 patients who were asked, 739 patients were willing to participate (40%). Reasons not to participate were: feeling psychologically incapable to participate (*n* = 539), not wanting to participate (*n* = 228), no time (*n* = 169) and feeling physically incapable to participate (*n* = 60); 71 patients provided no reason. In total, 262 informal caregivers agreed to participate. An overview of the sociodemographic and clinical characteristics of the 739 HNC patients and 262 informal caregivers is provided in Table 2.

**Table 2:**
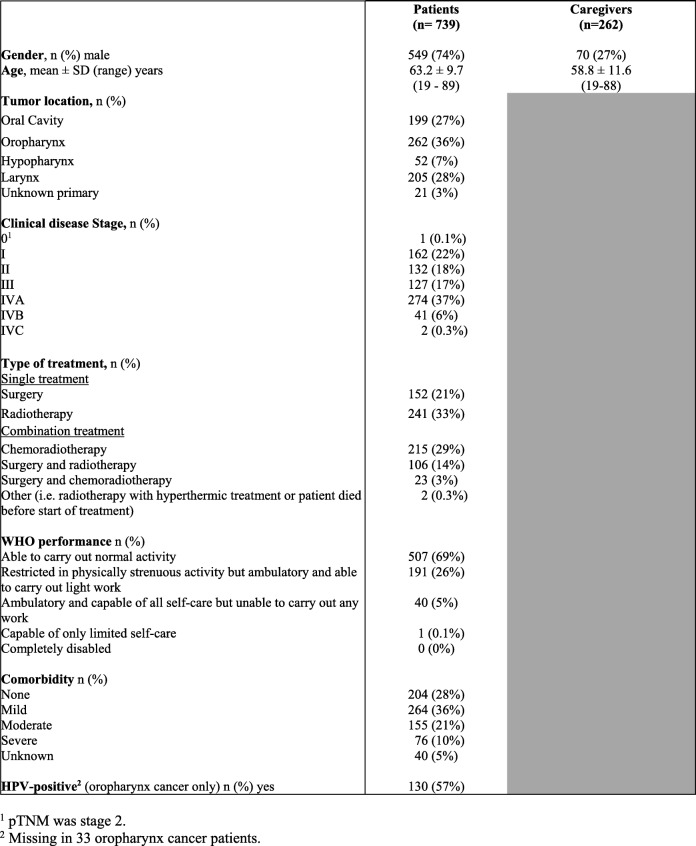
Overview of the characteristics of the NET-QUBIC population.

To evaluate possible bias in the NET-QUBIC cohort in relation to the patient cohort in the Netherlands, we obtained data from the Netherlands Cancer Registry (NCR) on the total Dutch HNC population in all eight HNC centers, treated from March 2014 to June 2018 in the Netherlands (the same study period as in the NET-QUBIC project) for newly diagnosed squamous cell carcinomas in the head and neck (oral cavity, oropharynx, hypopharynx, larynx, unknown primary; all stages); age > 18 years; all treatment modalities (surgery, radiotherapy, chemotherapy); exclusion criteria were: other tumors in the head and neck (e.g. lymphoma, skin malignancies, thyroid cancer). These are the same in- and exclusion criteria as in the NET-QUBIC project. The NCR does not have data on the NET-QUBIC inclusion criteria “treatment with curative intent; able to write, read, and speak Dutch” nor on the exclusion criteria “patients unable to understand the questions or test instructions; severe psychiatric co-morbidities (i.e. schizophrenia, Korsakoff’s syndrome, severe dementia); unable to understand informed consent”. The NCR does not have information either on informal caregivers of HNC patients. In total, we obtained overall data (sex, age, HNC subsite, stage, and treatment modality) on 9802 Dutch HNC patients.

The NET-QUBIC patient cohort (*n* = 729 (739–10 patients who did not provide informed consent to combine their data with other registries)) was compared to the total Dutch HNC patient population (*n* = 9073 (9802–729)) regarding sex, age, tumor subsite and stage, and treatment modality (Chi-square or t-tests, *p* < .01 was considered as significantly different) (Table 3). The NET-QUBIC cohort differed significantly from the Dutch HNC patient population regarding sex (less females (26% vs. 33%)), age (on average 3 years younger), tumor subsite (relatively less oral cavity cancer (27% vs. 36%) and more oropharynx cancer (36% vs. 24%)), and treatment modality (relatively less often surgery (38% vs. 44%), and more often radiotherapy (79% vs. 64%) and chemotherapy (32% vs 20%). There were no differences regarding tumor stage (Table 3).

**Table 3:**
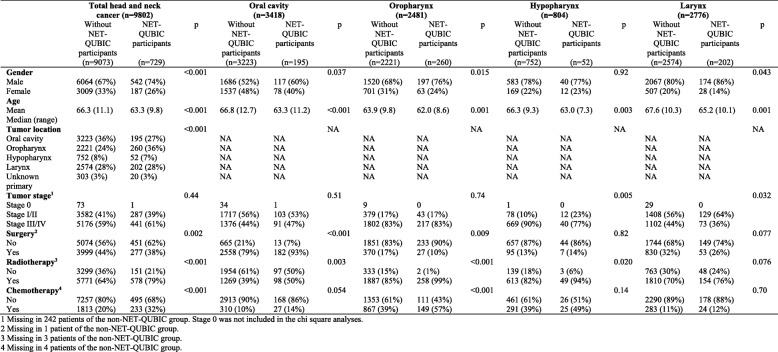
Differences in demographic and clinical characteristics between the NET-QUBIC HNC patient cohort and the general Dutch HNC patient population

We also compared the NET-QUBIC cohort with the Dutch HNC population per tumor subsite (oral cavity, oropharynx, hypopharynx, larynx) regarding sex, age, tumor stage, and treatment modality (Table 3). The NET-QUBIC oral cavity group was on average 3.5 years younger, and more often treated with surgery (93% vs. 79%) and radiotherapy (50% vs. 39%) compared to the total Dutch oral cavity cancer group. The NET-QUBIC oropharynx group was on average 2 years younger, less often treated with surgery (10% vs 17%), more often with radiotherapy (99% vs 85%), and more often with chemotherapy (57% vs 39%). The NET-QUBIC hypopharynx cancer group was on average 3 years younger, and more often diagnosed with early stage cancer (23% vs. 10%) compared to the total Dutch hypopharynx cancer group. The NET-QUBIC larynx cancer group was, besides on average 2 years younger, otherwise comparable with the total Dutch larynx cancer group.

## Discussion

Literature suggests that the course of HRQOL and its association with survival is influenced by various cancer-related, personal, genetic, biological, psychobehavioural, physical, lifestyle-related, and social factors, which may also interact with each other. Many (molecular) mechanisms possibly influencing the course of HRQOL and the relation between HRQOL and survival are unknown. There is a strong unmet need for high quality data and biological sample resources to advance our knowledge. Besides NET-QUBIC, there are a few other large resources such as national registries (e.g. the United States National Cancer Institute program [62], the Dutch Head and Neck Society [5], the Danish Head and Neck Cancer Group [63]), the data warehouse of the EORTC with data of international clinical trials (www.eortc.be), and some other large longitudinal cohort studies in the United States [64–66], and the Head and Neck 5000 (HN5000) project in the UK [67, 68]. These large resources can be seen as complementary, as they all have clinical and socio-demographic information, but some have biological samples and others do not, some have detailed data on symptoms, lifestyle, and HRQOL in a relatively smaller (< 1000, e.g. NET-QUBIC) cohort and others have less detailed data but in a relatively larger cohort (e.g. the HN5000), all dependent on the purpose and capacities of these registries and projects.

A strength of NET-QUBIC is that the outcome assessment protocol was developed following the standardized assessment and evaluation of functioning based on the International Classification of Functioning, Disability and Health (ICF) Core Set for HNC (www.icf-core-sets.org), and the protocol also complies with the HNC toolbox as described by Ringash et al. [69]. We followed a three step approach including 1) preselecting instruments for the assessment of HRQOL and functioning, based on our previous research targeting HNC patients and other large scaled cohort studies on ageing (www.lasa.nl) and depression and anxiety (www.nesda.nl), (2) a pilot study among 15 HNC patients which proved that the outcome assessment protocol is feasible [70], (3) finalizing the outcome assessment protocol and study procedures including SOPs and training for the field workers. However, not all selected outcome measures have undergone thorough evaluation of all psychometric criteria according to the COnsensus-based Standards for the selection of health Measurement INstruments (COSMIN) criteria (www.cosmin.nl). Another strength is the large longitudinal biobank that can be applied for tumor-educated blood platelet profiling (already carried out), circulating tumor DNA analysis (already carried out), and immune profiling, and the data linked to outcome and HRQoL.

Another strength of NET-QUBIC is that data and samples are collected according to FAIR principles: Findable, Accessible, Interoperable, Re-usable. Legal issues are handled through a multicenter research agreement including MTA/DTA, taking into account the GDPR. There were several problems encountered before we achieved this agreement: the laboratory infrastructure had to be set-up at some centers, and storage of samples to local protocols required reorganization of the planned biobanking. Also the procedures on making data and biosamples available had to be centralized to prevent a huge administration for the researchers, and this was not in line with the standard policies at the participating institutes. A consensus could be reached between all centers, but this required extensive preparations. These problems caused a delay in starting recruitment of participants in some centers and took considerable time and efforts of the (local) principal investigators and legal advisors during the study. A limitation of NET-QUBIC is the response rate of 40%, which is slightly lower than the response rate as reported in the HN5000 cohort (49%) [68]. The NET-QUBIC cohort is not completely representative for the Dutch HNC population regarding age (on average 3 years younger), sex (less women participated), tumor subsite (less patients with oral cavity cancer and more patients with oropharynx cancer), and treatment modality. There were no differences regarding tumor stage.

The NET-QUBIC Datawarehouse and Biobank are open for collaboration with (inter)national researchers. Already planned studies include 1) The course of HRQOL in HNC patients from baseline to 2 years follow-up, and to identify cancer-related, personal, genetic, biological, psychobehavioural, physical, lifestyle-related, and social determinants of HRQOL, 2) Whether the association between HRQOL and survival is direct or mediated by other variables, 3) The course of HRQOL in informal caregivers, 4) The course of symptoms of depression in HNC patients and the relation with survival, 5) Treatment optimization in elderly HNC patients, and 6) Predictive models for objectively and subjectively measured salivary, mastication, and swallowing function in relation to HRQOL. The first data release in 2017 granted access to 6 other studies making use of data of the first 254 included patients. The second data release in 2019 granted access to 7 new studies making use of all 739 included patients and 262 caregivers at baseline, and at 3 and 6 months follow-up. In the near future, new data releases will be organized and will be announced on the website www.kubusproject.nl.

In conclusion, by granting access to all interested researchers to the NET-QUBIC data, we enable new research lines in for example: clinical research (e.g. treatment optimization in elderly patients), biological research (e.g. liquid biopsy analysis for follow up), molecular/digital pathology based predictive biomarker research, HRQOL research (e.g. the impact of toxicity on HRQOL), and interrelated research (e.g. prediction of treatment response, based on clinically validated biomarkers; HRQOL in relation to biomarkers and survival). Managing this access in an integrated (inter)national network is crucial to advance research with broad, multidisciplinary impact.

## Data Availability

Data and materials are described on the NET-QUBIC project website (www.kubusproject.nl). The collection and integration of large amounts of personal, biological, genetic and diagnostic information precludes open access to the NET-QUBIC research data. In the section Data and sample dissemination is described how the data are made available for the research community.

## Abbreviations

ACE-27: Adult Comorbidity Evaluation-27
BPI: Brief Pain Inventory
CFQ: Cognitive Failure Questionnaire
CIDI: Composite International Diagnostic Interview
COSMIN: COnsensus-based Standards for the selection of health Measurement Instruments
CPI: Close Person Inventory
CRA: Caregiver Reaction Assessment
DTA: Data Transfer Agreement
eCRF: electronic Case Report Form
EDTA: EthyleneDiamineTetra Acid
EORTC: European Organization for Research and Treatment of Cancer
EQ-5D-3 L: The 5-level EuroQol 5 Dimension questionnaire
FAIR: Findable, Accessible, Interoperable, Re-usable
FROG: Functional Rehabilitation Outcome Scale (FROG),
FSFI: Female Sexual Function Index
GDPR: European General Data Protection Regulation
GES: Generalized self-efficacy scale
GRIX: Groningen Radiation Induced Xerostomia
HADS: Hospital Anxiety and Depression Scale
HN35: EORTC module Head and Neck 35
HN5000: Head and Neck 5000
HNC: Head and Neck Cancer
HPA: HypothalamicPpituitary-Adrenal
HPV: Human Papilloma Virus
HRQOL: Health Related Quality of Life
IADL: Instrumental Activities Daily Life
ICF: International Classification of Functioning, Disability and Health
IES-R: Impact of Event Scale-Revised
IIEF: International index of Erectile Function
IKNL: Netherlands Comprehensive Cancer Organisation
IL: InterLeukin
iMCQ: Medical Consumption Questionnaire
IPA: Impact on Participation and Autonomy
iPCQ: Productivity costs questionnaire
MAC: Mental Adjustment to Cancer scale
MDD: Major Depressive Disorder
MFI: Multidimensional Fatigue Inventory
MNA: Mini Nutritional Assessment
MTA: Material and data Transfer Agreement
NCR: Netherlands Cancer Registry
NEO-FFI: The NEO Five Factor Inventory
NET_QUBIC: NETherlands QUality of life and BIomedical Cohort
PASE: Physical Activity Scale for the Elderly
PBMC: Peripheral Blood Mononuclear Cell
PCR: Polymerase Chain Reaction
PROM: Patient Outcome Measure
PSMS: Pearlin & Schooler mastery scale
PSQI: Pittsburgh Sleep Quality Index
QLQ-C30: Quality of Life Questionnaire C30
SCNS-HNC: Supportive care needs survey- HNC-specific module
SCNS-P&C: Supportive care needs survey for partners and caregivers
SCNS-SF34: Supportive care needs survey – short form
SDQ: Shoulder Disability Questionnaire
SES: Socio-Economic Status
SHI: Speech Handicap Index
SOP: Standard Operating Procedure
SSL-I12: Social Support List Interactions
SwalQoL: Swallowing QoL Questionnaire
TNF-a: Tumor Necrosis Factor alpha
TNM: Tumor, Node, Metastasis
UCL: Utrecht Coping List
WHO: World Health Organization
