# Correction to: Advancing interdisciplinary research in head and neck cancer through a multicenter longitudinal prospective cohort study: the NETherlands QUality of life and BIomedical Cohort (NET-QUBIC) data warehouse and biobank

**DOI:** 10.1186/s12885-019-6223-y

**Published:** 2019-10-22

**Authors:** I. M. Verdonck-de Leeuw, F. Jansen, R. H. Brakenhoff, J. A. Langendijk, R. Takes, C. H. J. Terhaard, R. J. Baatenburg de Jong, J. H. Smit, C. R. Leemans

**Affiliations:** 1 Department of Otolaryngology-Head and Neck Surgery, Cancer Center Amsterdam, Amsterdam UMC, Vrije Universiteit Amsterdam, PO BOX 7057, 1007 MB Amsterdam, The Netherlands; 2 Department of Clinical, Neuro and Development Psychology, Vrije Universiteit Amsterdam, Amsterdam Public Health Research Institute, Amsterdam, The Netherlands; 3 Department of Radiation Oncology, University Medical Center Groningen, University of Groningen, Groningen, The Netherlands; 4 Department of Otolaryngology-Head and Neck Surgery, Radboud University Medical Center, Nijmegen, The Netherlands; 5 Department of Radiation Oncology, University Medical Center, Utrecht, The Netherlands; 6 Department of Otolaryngology and Head and Neck Surgery, Erasmus Cancer Institute, ErasmusMC, Rotterdam, the Netherlands; 7 Department of Psychiatry, Neuroscience Campus Amsterdam and Amsterdam Public Health Research Institute, Amsterdam UMC, location VU University Medical Center, Amsterdam, The Netherlands

**Correction to: BMC Cancer (2019) 19:765**

**https://doi.org/10.1186/s12885-019-5866-z**

Following publication of the original article [1], the authors reported the name of R.J. Baatenburg de Jong was incorrectly tagged in the HTML version of the article. The correct tagging is:

Given name: R (Robert)

Middle name: J

Family name: Baatenburg de Jong

The original article [1] has been corrected.
